# Directional genomic hybridization (dGH™) identifies small inverted duplications *in situ*

**DOI:** 10.3389/fgene.2025.1604822

**Published:** 2025-06-23

**Authors:** Thomas Liehr, Erin Cross, Stefanie Kankel

**Affiliations:** ^1^ Jena University Hospital, Friedrich Schiller University, Institute of Human Genetics, Jena, Germany; ^2^ Kromatid, Inc., Longmont, CO, United States

**Keywords:** small supernumerary marker chromosomes, molecular cytogenetics, inverted duplication, single-chromatid fluorescence *in situ* hybridization, directional orientation

## Abstract

Although fluorescence *in situ* hybridization (FISH) is a standard approach for characterizing the chromosomal structure involving a region of interest, FISH targeting single chromatids is not routinely performed. However, this latter approach seems principally well-suited to distinguish small, tandem inverted duplications from direct duplications in clinical cases. A commercially available single-chromatid FISH approach, called “directional genomic hybridization” (dGH™), was applied in this study to nine cases of small supernumerary marker chromosomes (sSMCs) known to contain inverted duplications. Successful detection of small inverted duplications has been demonstrated for the first time in this study using a custom Kromatid dGH™ InSite Assay. In all five euchromatic sSMC cases, inversions were detected using the dGH single-chromatid molecular cytogenetic assay. Thus, the dGH method of FISH is a readily applicable, straightforward approach for identifying small inverted duplications that are undetectable by conventional (molecular) cytogenetic methods. This technique may be used to identify the presence of small inversions within regions presenting a copy number gain as detected by chromosome microarray. Distinguishing small inverted duplications from direct duplications may have an impact on topologically associating domains (TADs) and, thus, on clinical outcome.

## 1 Introduction

The advent of chromosomal microarrays (CMAs) has enabled major advances in the detection of previously undetectable small deletions and duplications in the human genome (Martin et al., 2015). Such copy number variations (CNVs) in the range from kilobase to megabase pairs can be further characterized by fluorescence *in situ* hybridization (FISH) (Liehr, 2021). In this way, tandem duplications can be clearly distinguished from insertions elsewhere in the genome (Gu et al., 2016) and from the presence of a small supernumerary marker chromosome (sSMC) (Liehr et al., 2009) or a CNV resulting from a (more complex) chromosomal rearrangement (Pellestor et al., 2011). The influence of duplications on the formation of topologically associating domains (TADs) has been shown in recent studies. TADs are important in the regulation of DNA replication and transcription; TAD disruption can be due to submicroscopic rearrangements like an inversion, a deletion, and, specifically, a duplication (Lupiáñez et al., 2016; Franke et al., 2016; Rajderkar et al., 2023). These findings highlight the importance of characterizing and studying these events. However, standard FISH and other methods cannot easily determine whether a small duplication is in direct or inverted orientation, even though inverted duplications of this type have been linked to diseases like *retinitis pigmentosa* (de Bruijn et al., 2020).

Several FISH approaches are described in the literature with which single-chromatid signals can be obtained for repetitive sequences, such as telomeres (Goodwin and Meyne, 1993; Bailey et al., 2004; Williams et al., 2011; Rubtsov and Zhdanova, 2017). In these approaches, only one parental DNA strand per chromatid is labeled by a specific unidirectional DNA probe to obtain single-chromatid signals, and the daughter-strand DNA on the second chromatid must be degraded, preventing hybridization with the FISH probe unless an inverted structural variant or sister chromatid exchange is present. A commercially available method for directional single-chromatid hybridization of unique sequences—directional genomic hybridization (dGH) (Kromatid, Longmont, Colorado, United States)—was applied for the first time in this study to visualize the inverted and duplicated structure of inverted duplicated sSMCs at the chromosomal level in an established model system (Spittel et al., 2014; Liehr et al., 2023).

Carriers of an sSMC comprise a heterogeneous group, ranging from clinically healthy individuals to those who are severely affected. Worldwide, there are ∼3.3 × 10^6^ sSMC carriers. Of these, 70% are completely normal, apart from fertility problems in a small subgroup; thus, most sSMC carriers may never become aware of their condition. However, the remaining 30% show clinical symptoms and represent a mix of >100 rare diseases. Among the best known clinically significant conditions are a) +inv dup(12)(pter→q10∼12:q10∼12→pter)/tetrasomy 12p or Pallister–Killian syndrome (OMIM #601803); b) +inv dup(18)(pter→q10:q10→pter)/tetrasomy 18p syndrome (OMIM #614290); c) +inv dup(22)(pter→q11.2:q11.2→pter)/proximal tetrasomy 22q or cat eye syndrome (OMIM #115470); and d) +der(22)t(11;22)(q23;q11.2)/derivative chromosome 22 or Emanuel syndrome (OMIM #609029) [summarized by Liehr et al. (2023)].

sSMCs are simultaneously a structural and numerical aberration, and accordingly, they are an interesting object for chromosomic research (Liehr et al., 2023). sSMCs can originate from any of the 24 human chromosomes, be composed of material from one or more chromosomes, be continuous or discontinuous, have a regular centromere or a neo-centromere, and occur in various shapes (Liehr, 2023; Liehr et al., 2023). They can have centric minute, ring, or inverted duplication shapes. Most of them originate from an acrocentric chromosome, and according to the literature, up to 70% of sSMCs derive from chromosome 15 (Liehr, 2023). Most *de novo* sSMCs are believed to result from incomplete trisomic rescue (Liehr, 2018), which may include chromothripsis events. In addition, 30% of sSMCs are inherited within families, sometimes over many generations (Liehr, 2023).

Due to their nature, sSMCs are best detected by GTG banding and further characterized by FISH. As they tend to be mosaic and many of them are exclusively heterochromatic, methods like CMA tend to miss up to 80% of this type of cytogenetic aberration (Liehr and Hamid Al-Rikabi, 2018). Although molecular cytogenetic approaches are available for characterizing sSMC origin and content (Liehr, 2023), there was no way to prove or disprove the inverted duplication nature of sSMCs using FISH. In this study, a custom dGH™ InSite Assay, comprised of targeted probes for the regions of interest, was used to analyze nine sSMC cases from the Else Kröner–Fresenius sSMC cell bank (https://cs-tl.de/DB/CA/sSMC/33-EKF/a-Start.html). The suitability of this approach to characterize the orientation of a small inverted duplication event was investigated for the first time in this study.

## 2 Materials and methods

### 2.1 Cell lines and their work-up for dGH™

Nine cell lines from the Else Kröner–Fresenius sSMC cell bank (https://cs-tl.de/DB/CA/sSMC/33-EKF/a-Start.html) were used, as listed in Table 1. The sSMCs were previously characterized by FISH (Spittel et al., 2014) and are described according to ISCN (2020). Five cell lines had one or two identical sSMCs derived from chromosome 15, three carried an sSMC (22), and one carried an sSMC (21); four of them were purely heterochromatic.

**TABLE 1:** List of nine B-cell lymphocyte lines used in this study, their karyotype, and the structure of the sSMCs.

Number	Cell line name	Karyotype	sSMC
1	13L0187/EKF-#15-q11.1/14-i	mos 47,XY,+mar[63]/46,XY[1]	inv dup(15)(q11.1)
2	12L0112/EKF-#15-q11.1/3-i	47,XX,+mar[99]/46,XX[1]	inv dup(15)(q11.1)
3	12L0141/EKF-#15-q12/1-i	47,XY,+mar[100]	inv dup(15)(q12)
4	12L0131/EKF-#15-q14/1-i	47,XX,+mar[49]/46,XX[1]	inv dup(15)(q14)
5	11L369/EKF-#15-q14q13.2q13.3/1-i	48,XY,+marx2[50]	der(15)(pter→q13.1∼13.2::q13.3→ q14::q14→q13.3::q13.1∼13.2→pter)
6	12L0092/EKF-#21-q11.2/1-i	47,XY,+mar[149]/46,XY[1]	inv dup(21)(q11.2)
7	2008B246/EKF-#22-q11.1/4-i	47,XX,+mar[51]	inv dup(22)(q11.1)
8	12L0109/EKF-#22-q11.1/8-i	47,XY,+mar mat[50]	inv dup(22)(q11.1)
9	05PO209/EKF-#22-q11.21/1-i	47,XX,+mar[48]/46,XX[2]	inv dup(22)(q11.21)

The cell lines were grown in RPMI-1640 medium with glutamine supplemented with 15% fetal bovine serum and antibiotics (penicillin/streptomycin) (Weise and Liehr, 2017). To enable single-chromatid-directed FISH, the following protocol was used: each of the nine B-cell lymphocyte lines (Table 1) was thawed and cultivated until confluent. The cell culture medium was replaced with fresh culture medium containing nucleotide analogs BrdU/BrdC (dGH cell prep kit, Kromatid, Longmont, Colorado, United States, Cat# dGH-0001) for 18 h. Cells were arrested in the first mitosis with a 1.5-h colcemid block (Kromatid, Longmont, Colorado, United States, Cat# COL-001), harvested, and fixed in freshly made fixative (3:1 methanol: acetic acid). Metaphase spread preparation and subsequent UV and exonucleolytic treatments were performed to selectively remove the analog-incorporated daughter strands according to published dGH protocols (Ray et al., 2013). Standard chromosome preparation was then carried out from the cell cultures of the B-lymphocytes (Weise and Liehr, 2017).

### 2.2 Probe selection for dGH™

A custom dGH InSite assay, including three unique sub-centromeric probes, was performed on the samples. Sub-centromeric probe targets included the following genomic regions:

- subCEP CHR 15q in 15q11.2, chr15:25,742,858–27,215,190 (GRCh38);
- subCEP CHR 21q in 21q11.1∼21.1, chr21:13,299,000–16,295,000 (GRCh38);
- subCEP CHR 22q: in 22q11.1∼11.21, chr22:15,844,000–19,177,000 (GRCh38).

A standard FISH experiment was conducted on the chromosome preparations described in Section 3.1.

## 3 Results

**FIGURE 1:**
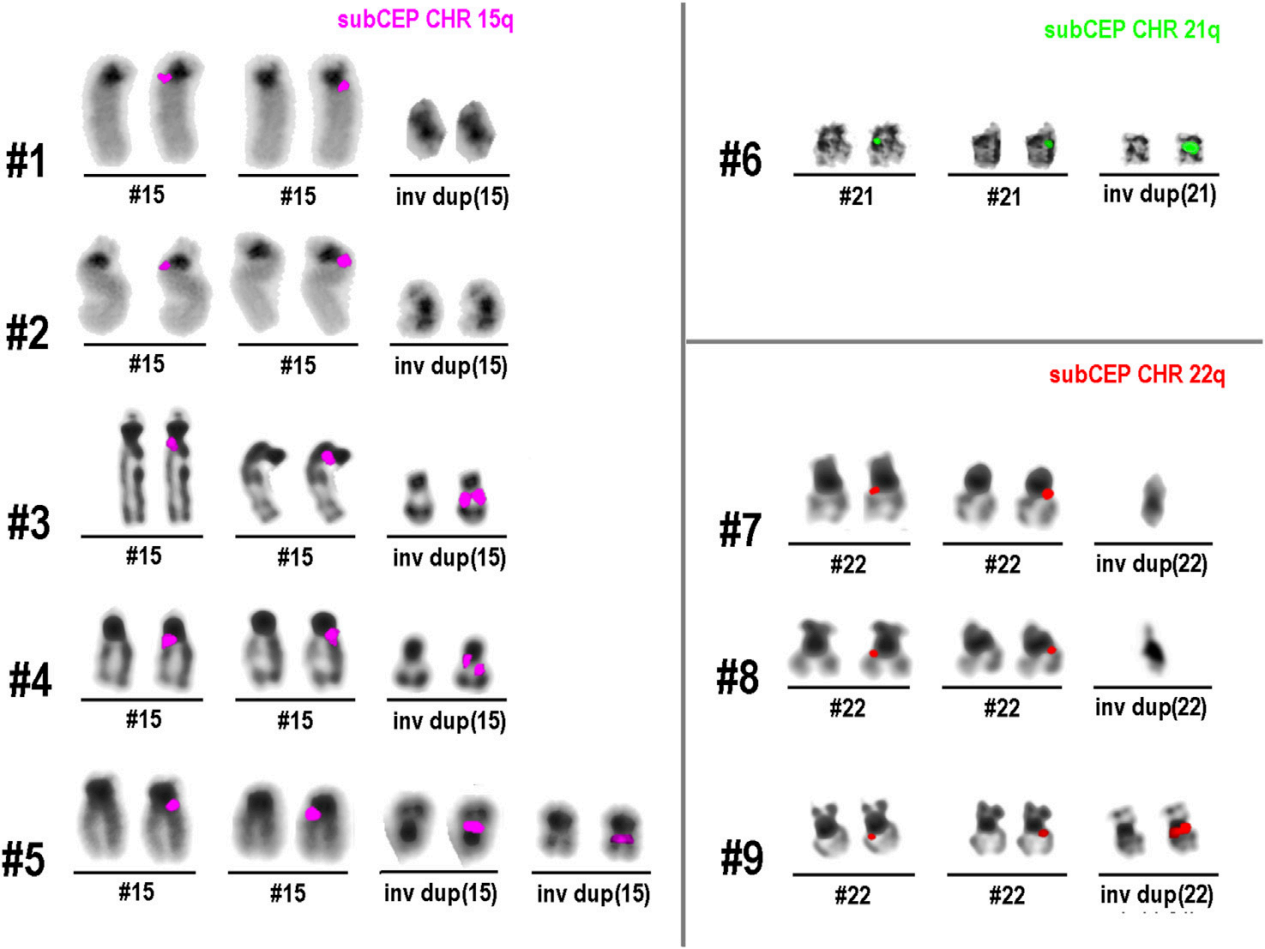
Results of single-chromatid FISH (dGH)™ obtained in nine sSMC cases listed in Table 1. All normal chromosomes show only one signal in one chromatid, while all euchromatic sSMCs (cases 3–6 and 9), due to their inverted duplicated structure, show signals on both chromatids.

All nine cases with an inverted duplication-shaped sSMC (see Table 1) were hybridized with the suited chromosome-specific probes. The obtained results are shown in Figure 1. In all cases, the normal chromosome pairs for 15, 21, or 22 each showed only a single-chromatid signal. In cases 1, 2, 7, and 8 with heterochromatic sSMCs, the probes used did not span regions present on these derivative extra chromosomes; therefore, there were no detectable signals on them. In each of the other five cases, there were specific double-chromatid signals on the inverted duplicated sSMCs, confirming their inverted duplication shape.

## 4 Discussion

sSMCs and their analysis in diagnostics represent an established approach (Liehr, 2023). In this study, inverted duplicated sSMCs were analyzed for the first time using single-chromatid FISH. The results show that this approach can provide visual, easy-to-interpret information on the orientation of small, closely co-localized DNA segments. In all five cases of euchromatic sSMCs, the reverse orientation of the duplicated segments clearly showed up as double-chromatid signals rather than single-chromatid signals on the sSMCs. This is the expected result when two closely adjacent DNA segments are aligned in opposite orientations. In the case of direct—or tandem—duplication, a double signal would be visible on only one of the two chromatids. The signal would appear similar to that of the normal chromosomes shown in Figure 1: a signal on only one chromatid, with the duplicated region appearing more intense.

In research, sSMCs and other chromosomal rearrangements with duplications often pose a challenge (Liehr, 2021; Liehr, 2023). Although in an sSMC, its inverted duplicated nature can be implied by banding cytogenetics, this is not valid in many other cases with intrachromosomal duplications. Considering that inversions combined with duplications may contribute to significant changes in gene expression (as noted in the *Introduction*), this research demonstrates that the directional information provided using the Kromatid dGH™ method offers a versatile approach for investigating such rearrangement.

The Kromatid method of single-chromatid FISH (dGH™) provides an advanced cytogenetic solution for characterizing sSMCs, in addition to intrachromosomal duplication events, and can be considered a new and easy-to-use approach to solve questions on the orientation of small chromosomal rearrangements. Yet, the bottleneck in single-chromatid FISH approaches has been the (non-)availability and high cost of suited DNA probes (Goodwin and Meyne, 1993; Bailey et al., 2004; Williams et al., 2011; Rubtsov and Zhdanova, 2017). With a commercial option offering a wide range of DNA probes for single-chromatid FISH, individual cases with small CNVs as gains from copy numbers are also accessible in this way.

In conclusion, the present study has shown that small inverted duplications can be clearly ascertained by directional genomic hybridization (dGH)™. In addition to this application, the model system of “inverted duplicated sSMCs” will also be suitable for determining the orientation of small tandem duplications. The latter are of particular interest as the clinical impact of the orientation of tandem duplications has not been accessible so far but could influence the effects on TADs (Rajderkar et al., 2023).

## Data Availability

The original contributions presented in the study are included in the article/supplementary material; further inquiries can be directed to the corresponding author.
